# The role of workday characteristics on perceived stress and time pressure among nurses in Finnish long-term care – a cross-sectional study

**DOI:** 10.1186/s12913-024-11294-4

**Published:** 2024-08-02

**Authors:** Visa Väisänen, Salla Ruotsalainen, Laura Hietapakka, Juhani Sulander, Timo Sinervo

**Affiliations:** 1 Finnish Institute for Health and Welfare, Welfare State Research and Reform unit, Health and Social Service System Research team, Mannerheimintie 166, Helsinki, 00330 Finland; 2 Faculty of Social Sciences and Business Studies, Department of Health and Social Management, University of Eastern Finland, Kuopio, Finland

**Keywords:** Long-term care, Care workforce, Job demands, Nurse, Stress, Strain, Time pressure, Workday characteristics

## Abstract

**Background:**

Aging populations and nursing workforce issues are causing challenges for long-term care globally, and therefore, improving the work-related wellbeing and retention of nurses is crucial. As such, gaining a further understanding of the factors that affect work strain in long-term care is important. Previously, the effect of job demands on the wellbeing of nurses has been researched principally by subjective instruments. In this study, we examined the relationship between indirectly measured workday characteristics and perceived stress and time pressure among nurses working in Finnish long-term care (assisted living facilities with 24-hour assistance).

**Methods:**

A total of 503 nurses from 44 assisted living facilities across Finland completed time measurement surveys and wellbeing questionnaires. The data were linked with client characteristics from the Resident Assessment Instrument register. The relationships between the measured number of care events during the workday, clients’ care needs, and the amount of breaktime and perceived stress and time pressure were analyzed using multivariate logistic regression.

**Results:**

Nurses who had more care events and clients with greater care needs were at higher odds of having high stress. More care events and reduced breaktime were associated with high time pressure. Disruptions during the workday were strongly associated with both high stress and time pressure. Last, nurses who were under high stress and time pressure worked more often in teams with lower team autonomy.

**Conclusions:**

Our findings on indirectly measured job demands indicate that dividing the workload equally among nurses through better work division can help reduce the stress and time pressure of nurses in long-term care. In addition, ensuring sufficient breaktime and preventing unnecessary disruptions is important. To help recruit and retain the care workforce, fair management of work that accounts for varying client care needs and workload is needed. In addition, legislative and governance tools, such as staffing level regulation, and further consideration of job demands might aid in reducing the job strain of nurses.

**Patient or public contribution:**

Patients or nurses were not involved in the design of the study, analysis, or interpretation of the results, or in the preparation of the manuscript.

**Supplementary Information:**

The online version contains supplementary material available at 10.1186/s12913-024-11294-4.

## Background

Aging populations are causing challenges for both health and social care globally, and the costs of long-term care are expected to increase substantially in the next decades [1, 2]. Another established challenge is cognitive decline, which affects an increasing number of Finnish long-term care clients [3, 4]. This is relevant, as caring especially for dementia clients might be more straining for nurses [5] and higher physical workload has been reported among nurses working with dementia clients [6]. Furthermore, as older clients with cognitive decline appear to have significantly higher care needs compared to clients without cognitive decline, which can be attributed to mobility disability and lower physical functioning [4], nursing work might become increasingly straining in the future. Simultaneously, the current shortage and growing need of care workforce [7] has further exacerbated the issues of care for older people. In order to sustain long-term care services, increasing the work-related wellbeing, retention, and recruiting of nurses and care workforce is paramount [2, 8].

Formal long-term care workers, especially nurses, are in a key role in long-term care, providing assistance with activities of daily living and facilitating the pharmacotherapy and medical care needed [8]. In Finland, long-term care is mostly provided by licensed practical nurses and in a smaller part by registered nurses. Nursing work is often characterized as stressful and demanding. Previous studies have demonstrated high efficiency demands and poor work-related wellbeing for nurses working in long-term care for older people [9], which have been associated with higher intentions to leave [10], increased sickness absences [11], and lower quality of care [12]. In addition, a cumulation of different work stressors has been linked with reduced work ability [13]. Varying team models, higher team autonomy, and work wellbeing interventions have been suggested as possible responses to the problem [14, 15]. For example, previous research suggests that nurses working in teams with higher team autonomy have lower turnover intentions [16] and higher job satisfaction [17], mainly through lower perceived job demands and strain.

Unfortunately, it seems that working conditions or nurses’ wellbeing in long-term care are not improving. The wellbeing of nurses worsened significantly during the pandemic [18] and has not yet shown signs of recovery. Instead, many nurses are leaving care work and the workforce situation remains alarming [8]. In Finland, due to population aging and the high number of retiring care workers, the need for nurses remains substantial in the coming years: based on some estimations over 200 000 new nurses are needed by 2035 [19]. Further changes in the population structure also mean that an increasing number of clients have higher care needs, which require more care resources.

**Fig. 1:**
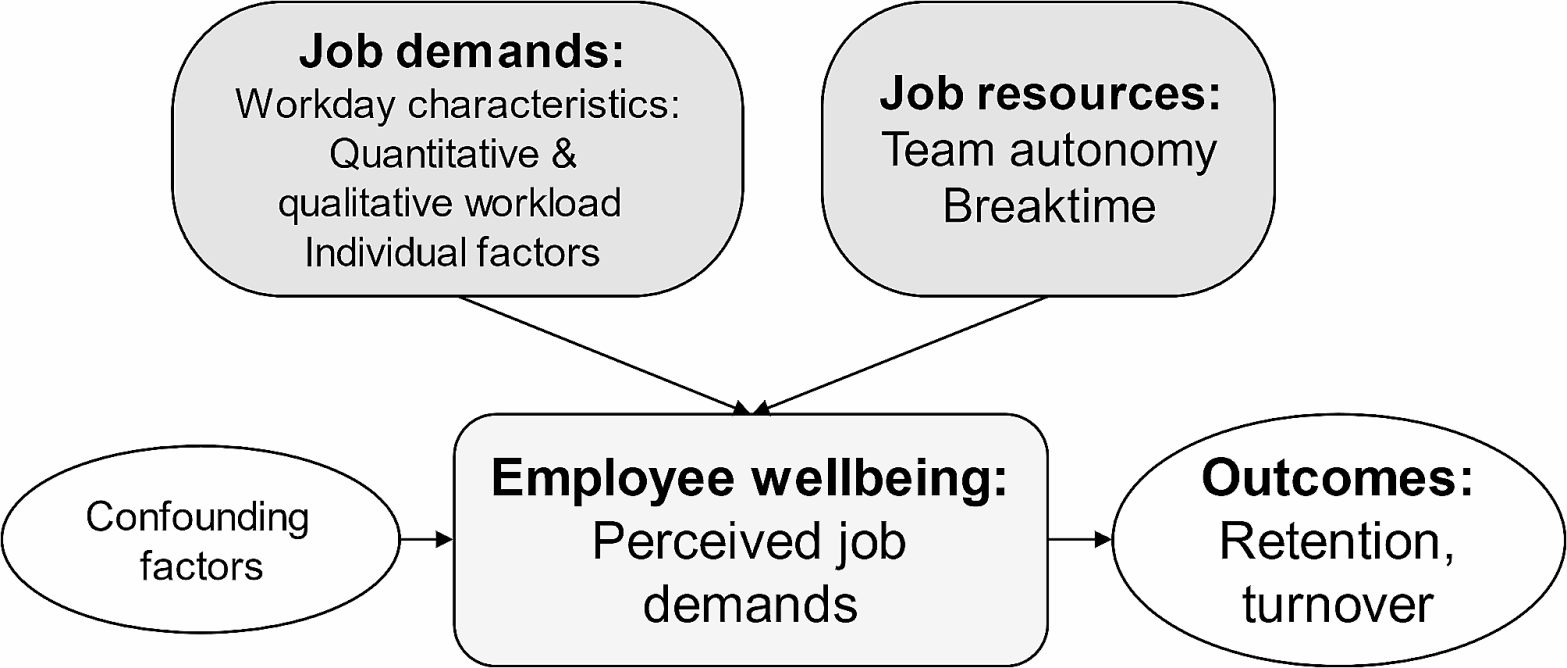
Conceptual framework of the study

The Job Demand-Resources (JD-R) model [20], which defines job demands as physical, psychological, social, or organizational in nature, was adopted as a theoretical framework for the study. Among nurses, high workload is a typical job demand, which is often studied as perceived time pressure. Instead of measuring subjective experienced workload, by using time allocation methodology we were able to investigate the workload indirectly. Employees reported what they did and which client they were caring for, which allowed for separately collected information on the workload, namely the number of care events, care needs of the clients, and amount of breaktime during the workday. These measures are relatively easy to construct and represent the workload through both a quantitative (care events) and a qualitative (care needs) view. Breaktime combines job demands and job resources, as adequate breaks require that the demands (clients to take care for) and the resources (time allocated) of the work are in relative balance. Regarding the job resources, we also studied organizational level indicators. Team autonomy has been increasingly studied in services for older people as the Dutch Buurtzorg model has reached positive outcomes [21]. Self-organized teams have very high autonomy in planning their work and in decision-making and sometimes have no frontline managers. While professional autonomy has been widely associated with better work environments and wellbeing outcomes, including lower stress [22], team autonomy, however, has mixed evidence [17, 23], which might be due to varying teamwork skills or management practices. The main study constructs and the assumed relationships are presented in the conceptual framework below (Fig. 1).

Previous studies on the job demands and wellbeing of nurses have mainly focused on subjective measures as explanatory variables, such as perceived workload or perceived stress [9, 24]. Meanwhile, research on objectively or indirectly measured workday characteristics and their relationship with stress or time pressure among nurses is scarce. Consequently, there remains a need to examine which common job demands (workday characteristics), such as the number of daily care events, clients’ care needs, and disruptions, in addition to job resource factors (work organization), such as team autonomy and size, are associated with the work-related wellbeing of nurses, when independently or indirectly measured. Further evidence on these factors can confirm previous research focusing on subjective instruments, help care units measure, design, and improve daily work organization and fair work division, and ultimately support retention and recruitment of nurses working in long-term care.

The aim of this study was to examine the effects of workday characteristics (care events, client care needs, break time) and work organization factors (team autonomy, team size) on perceived stress and time pressure among nurses working in Finnish long-term care (assisted living facilities with 24-hour assistance).

## Methods

### Design

The study was observational and cross-sectional in design.

### Study setting

Finnish long-term care for older people is mainly divided into a priority option of home care (combined home help and home nursing services) and assisted living facilities with 24-hour assistance aimed at clients with higher care needs [25]. Care is provided as a needs-based public service with relatively high user fees [25]. As of 2022, among the population aged 75 and over, 32% were home care clients and 7.3% were residents in assisted living facilities with 24-hour assistance [26, 27]. Care for older people is mainly provided by licensed practical nurses, who constitute approximately 70% of the workforce in assisted living [28]. Licensed practical nurses are social and healthcare professionals with three years of curriculum-based vocational education. Registered nurses (with a tertiary education) also have an important role in long-term care. Compared to acute or short-term care, the prevalence of licensed practical nurses is significantly higher in care for older people, where they have an independent responsibility for the care of older people. In other care settings, practical nurses often have a more assistive role and registered nurses work more closely with physicians [29].

In 2018–2019, the poor quality of care of Finnish assisted living facilities received a notable amount of media attention. Consequently, staffing level legislation was introduced in 2020, which mandated the number of nurse personnel per client [30]. Currently, the number is 0.65 nurses per client, but according to the government plans [31] it is expected to decrease to 0.60 by the end of 2026 with the aim of reducing costs. Previously, the legislation had received criticism as personnel requirements could not be fulfilled under the current workforce shortage and recruitment difficulties. In addition, the legislation might have had negative effects on home care, where the number of client visits decreased substantially in 2022, despite the aging population [27]. However, the current plans of lowering the staffing level have also faced resistance, especially from the worker’s unions [32].

### Data collection

Our study was part of a staff time measurement study conducted by the Finnish Institute for Health and Welfare in October 2021. The objectives of the project were to track the worktime division of employees in Finnish long-term care, to investigate the care services and care time given to the clients, and to examine which factors are associated with the wellbeing and retention of nurses.

Care units were recruited on a voluntary basis from the Finnish RAI (Resident Assessment Instrument) benchmarking network, which in 2021 covered approximately 52% of over 74-year-old clients in assisted living [33]. The use of RAI became mandatory in services for older people in 1.1.2023, and previously it was voluntary. The users were part of the RAI benchmarking network, which consisted of codevelopment and regular seminars [34]. In the end, 44 assisted living facilities (with 24-hour assistance) signed up for the study. The units were located in 15 out of 22 different wellbeing service counties (currently responsible for arranging health and social services) and in both rural and urban areas. Both public and privately owned care units participated in the study.

The number of surveys sent was based on an estimation of the number of employees working daily (information provided by the managers), as the precise number of employees working in the care units was not available. The estimated number of employees working daily at the participating work units was 917. In total 768 nurses returned the surveys, leading to an approximate response rate of 83.8%. However, the response rate includes uncertainty, as sickness absences, temporary workforce, and recruitment challenges might have affected the number of employees working.

Employees of Finnish long-term care units participating in the study documented their worktime using paper surveys developed for this study, including the start and end times of care tasks and other work, and the name of the client. The study period was one day (24 h), in accordance with previous staff time measurement studies, where 24-hour study periods are commonly used and are deemed adequate [35]. An optional brief wellbeing survey was included, which had one section of questions to be evaluated before the workday (items on sleep quality and recovery) and another set of questions for after the workday, with items relating to disruptions during the workday and perceived work-related wellbeing. To obtain information about client care needs, the survey data were merged with the Finnish RAI register, which includes standardized individual-level data on clients’ functioning, health status, and service needs [36]. In addition, an electronic survey for the managers of participating care organizations was sent, which included questions on the size of the organization and on the autonomy of care teams.

The questionnaires used in the study are presented in Supplementary file 1.

### Inclusion criteria

Study participants were limited to registered nurses and licensed practical nurses. To exclude nurses working outside of direct care, the participants were required to have a minimum of five clients during the day and a minimum of 15% of worktime spent on direct care (care work where the client was present). These criteria excluded 33 practical nurses and 17 registered nurses.

Last, to address missing data, participants with missing and nonmissing values in stress or time pressure were compared with the chi-squared test and the Kruskal-Wallis U test. Participants with missing values in the outcome variables did not differ statistically significantly from those with valid values. However, it appeared that participants with missing values could be slightly younger in age (stress: 44.3 and 39.4, *p* = 0.080, time pressure: 44.4 vs. 40.2, *p* = 0.072). In addition, participants with missing values in stress might have had clients with higher care needs (102.3 vs. 104.6, *p* = 0.067). Ultimately, participants with missing values in the outcome variables were excluded from the analyses (*n* = 44). To maximize the dataset, missingness in the other variables was handled using pairwise deletion (Variance: 0–28, mean = 7.4).

### Study measures

Outcome variables were retrieved from the second part of the wellbeing survey, which was completed after the workday. The outcome variables measured daily perceived strain, experienced during the workday. The first outcome variable stress was measured with a single-item question: *“Did you feel any stress today? (Stress means the situation when a person feels tense*,* restless*,* nervous*,* or anxious)”* [37].

The second outcome variable, time pressure, was measured with a two-item scale, adapted from the nurse stress index [38]. The participants were asked if the following had disturbed, worried, or strained them today. The two statements were *“I had too little time for my patients/clients”* and *“I did not have time to perform my work properly”*, which had a Spearman-Brown statistic of 0.81.

Both stress and time pressure were measured using a five-point Likert scale, from 1 *“Not at all”* to 5 *“Very much”*. The dependent variables of stress and time pressure were grouped into binary variables, as they were both strongly positively skewed. Answer options 1 *“Not at all”*, 2 *“Only a little”*, and 3 *“Somewhat”* were defined as low stress or low time pressure, and options 4 *“Quite much”* to 5 *“Very much”* were defined as high stress or high time pressure. Similar categorizations have been used in previous studies [39, 40].

There were three independent variables of interest, measured indirectly through the contents of the time measurement surveys the nurses filled during their workday. First, the number of care events during the day was calculated by the number of direct or indirect care tasks throughout the workday, which included a client. The variable correlated strongly with the amount of direct care time and was thus also used as a proxy variable for the general intensity of care work.

The care needs of the clients during the day were measured using the Case Mix Index (CMI) information from the clients’ RAI-assessments. The value was calculated as a mean of all care events that included a valid client’s name and thus a CMI-value. CMI is based on the client’s Resource Utilization Group (RUG), which is determined from individual clinical care needs and physical functioning. The index value corresponds to the care needs of the client, measured in cost adjusted care time needed, compared to the average client. For example, a client with a CMI value of 1.2 needs approximately 20% more care resources (care time) compared to an average client [41].

Breaktime was determined from the amount of breaktime in minutes that nurses documented in the time measurement forms. Lunch or dinner, coffee breaks, and other breaks were counted as breaktime. Coffee breaks or other social activities that were spent with clients were not considered as breaktime.

In addition, two adjusting variables were used from the wellbeing survey. First, recovery from work-related strain, which was answered before the workday began, was measured with the question *“Have you recovered from the strain caused by the previous workday?”*. Adequate recovery from work is essential for replenishing from strain and fatigue [42, 43], and increased job demands have been recently associated with lower recovery [44]. It was rated on a 10-point scale from 1 *“Not at all”* to 10 *“Very well”*. Due to the strongly negatively skewed distribution, the item was grouped into three categories: poor recovery (values 1 to 5), medium recovery (values 6 to 8), and good recovery (values 9 and 10). In addition, to adjust for the disruptions occurring during the workday, a question on how the workday went was used. Interruptions and disruptions of work are very common in nursing [45] and have been associated with negative employee wellbeing [46]. The question included three possible answers: *“[Workday] went as planned”*, *“[Workday] went nearly as planned”*, and *“Something disrupted the course of the workday”*. The item was dichotomized with the first two answers as the reference group.

Organization level data included the team size measured through the number of clients as indicated by the manager of the organization. In addition, team autonomy was measured as a sum variable of five items (Cronbach’s alpha: 0.70). The items concerned whether the team was able to make independent decisions regarding work shifts, division of care work tasks, recruitment of new employees, use of substitute workers, and participation in training. The questions were rated on a four-point Likert scale, from 1 *“Not at all”* to 4 *“Team is able to make decisions autonomously”*.

Last, background variables of age, sex, occupation, and work organization ownership (public or private) were used as adjusting variables.

### Statistical analysis

Demographics and workday characteristics of the study participants were explored using descriptive statistics. Percentages were used for categorical variables and means, and standard deviations were used for continuous variables. Statistical analysis was performed in two steps. First, to investigate the univariate associations, the Mann-Whitney U test was used to determine the statistical significance in ranks of independent variables (both individual and organizational factors) and the groups of the outcome variables of stress and time pressure. As the independent variables were not normally distributed and some of the variances were unequal between groups (as indicated by Fligner-Killeen’s test), a nonparametric test (Mann-Whitney U test) was used.

Second, to adjust for background variables, multivariate logistic regression was used to analyze the associations between the independent variables and the outcome variables. Multicollinearity was checked with VIF values (max: 1.16). Two models were built, one for low/high stress and one for low/high time pressure. All independent variables were entered in the model simultaneously, and the models were adjusted for background variables, including age, sex, occupation, organization ownership, recovery, and something disrupting the workday. The associations between the variables and the outcome were examined using odds ratios (ORs). The models were evaluated and compared using the C-statistic, with higher values indicating better discriminative performance of the model. Statistical significance was defined as *p* < 0.05. R version 4.2.2 was used for the statistical analyses [47].

It is important to note that the odds ratios reported correspond to a one-unit change in the continuous independent variables, which were one more care event per day, 1% higher average care needs of clients, and one minute more breaktime. To examine the effects of larger changes, odds ratios for larger units of change were calculated (10 more care events, 10% higher care needs, and 10 more minutes of breaktime).

## Results

In total, 503 nurses were included in the study (Table 1). The majority of the participants were licensed practical nurses and female. Stress and time pressure were rated on average 2.5 and 2.6 respectively, on a scale of 1 to 5. Nurses had on average 23 care events per day (SD: 13.3, min: 5, max: 86), and clients with a mean CMI value of 1.03 (SD: 0.07, min: 0.74, max: 1.16). The average breaktime per day was 28 min (SD: 19.2, min: 0, max: 221). Recovery from previous workday’s strain was reported on average as 7.8 (scale: 1–10), with 26% of participants having poor recovery (values 1 to 5). While the majority of the respondents had no disruptions in their workday, 23% of nurses reported disruptions in their workday.

**Table 1:** Participant characteristics (*n* = 503)

	All *n* = 503			
**Variable**	**N**	**%**	**Mean**	**SD**
**Age**			44.4	13.0
**Sex**,** female (%)**	467	93.6		
**Sex**,** male (%)**	32	6.4		
**Registered nurse (%)**	76	15.1		
**Practical nurse (%)**	427	84.9		
**Working in a public organization (%)**	336	67.0		
**Working in a private organization (%)**	166	33.0		
**Stress (1–5)**			2.48	1.21
High stress (4–5) (%)	106	21.1		
Low stress (1–3) (%)	397	78.9		
**Time pressure (1–5)**			2.58	1.13
High time pressure (4–5) (%)	136	27.0		
Low time pressure (1–3) (%)	367	73.0		
**Number of care events during the day**			22.7	13.3
**Mean CMI of clients**			1.03	0.07
**Breaktime (minutes)**			27.5	19.2
**Recovery (1–10)**			7.30	2.30
Recovery, good (9–10) (%)	167	34.7		
Recovery, medium (6–8) (%)	190	39.5		
Recovery, poor (1–5) (%)	124	25.8		
**Workday disruptions**				
Day went as planned (%)	364	76.6		
Something disrupted the workday (%)	111	23.4		

CMI: Case Mix Index

SD: Standard Deviation

A comparison of work characteristic means between low and high stress and time pressure participants are presented in Table 2. In total, 21% of the sample reported high stress (*n* = 106) and 27% reported high time pressure (*n* = 136). A small number of participants reported both high stress and high time pressure (12%, *n* = 59).

Participants who reported high stress had a statistically significantly higher mean number of care events during the day compared to participants reporting low stress (26 vs. 22). Those in the high time pressure group also had higher mean number of care events (25 vs. 22) and significantly lower breaktime (24 vs. 29 min) than those in low time pressure group. The mean CMI of the clients did not differ between participants with low and high stress or time pressure.

For the organizational variables, our results suggest that care workers with high stress and high time pressure work more often in organizations with lower team autonomy (*p* < 0.001 and *p* = 0.026 respectively). While team size measured in terms of the number of clients seemed to not have a relationship with stress, the results indicate that care workers in larger teams might work more often under high time pressure (*p* = 0.051), even though the result was not statistically significant.

**Table 2:** Mann-Whitney U test statistics for stress and time pressure and the independent individual and organizational variables

	Stress			Time pressure		
	**Low** *n* = 397	**High** *n* = 106		**Low** *n* = 367	**High** *n* = 136	
**Variable**	**Mean**		**p-value**	**Mean**		**p-value**
**Individual variables**:						
Number of care events during the day	**21.9**	**25.8**	**0.002**	**21.9**	**24.8**	**0.013**
Mean CMI of clients	1.02	1.03	0.218	1.02	1.03	0.365
Breaktime (minutes)	28.0	25.8	0.436	**28.7**	**24.4**	**0.036**
**Organizational variables**:						
Team size (clients)	25.6	26.2	0.501	25.5	26.3	0.051
Team autonomy (1–5)	**2.53**	**2.37**	< **0.001**	**2.52**	**2.44**	**0.026**

CMI: Case Mix Index

**Bold**: Mann-Whitney U test indicated statistically significant differences in means between low and high stress/time pressure groups

The results of the fully adjusted multivariate logistic regression can be seen in Table 3. The results indicate that a higher number of care events during the day (OR: 1.03, 95% CI: 1.01–1.05) and clients’ higher care needs (OR: 1.05, 95% CI: 1.00-1.09) were associated with nurses’ increased odds of being under high stress in assisted living facilities with 24-hour assistance. Furthermore, an increased number of care events per day (OR: 1.03, 95% CI: 1.01–1.04) and less breaktime (OR: 0.98, 95% CI: 0.97-1.00) were statistically significantly associated with participants being more likely under high time pressure.

Of the adjusting variables, poor recovery from work (OR: 4.91, 95% CI: 2.48–10.06) and something disrupting the workday (OR: 5.62, 95% CI: 3.25–9.89) were strongly associated with being under high stress or under high time pressure. Older age (OR: 1.02, 95% CI: 1.00-1.04) and something disrupting the workday (OR: 2.58, 95% CI: 1.57–4.23) were associated with increased odds of being under high time pressure. Other background variables were not statistically significantly associated with either outcome variable.

To further explore the effect sizes of the independent variables on stress and time pressure, odds ratios were calculated with larger values of change, comparable to possible meaningful changes in work characteristics. A change of 10 care events per day was associated with 34% higher odds of high stress and 30% higher odds of high time pressure (stress OR: 1.34, time pressure OR: 1.30). Similarly, a change of 10% in the mean CMI of clients increased the odds of high stress by 56% (OR: 1.56), and a change of 10 min in breaktime decreased the odds of high time pressure by 16% (OR: 0.84).

**Table 3:** Results of the multivariate logistic regression, adjusted for background variables

	Model 1: High stress		Model 2: High time pressure	
Variable	OR	95% CI	OR	95% CI
Age	0.99	0.97–1.01	1.02*	**1.00**–**1.04**
Female (ref.)				
Male	0.50	0.11–1.59	0.86	0.30–2.18
Practical nurse (ref.)				
Registered nurse	1.38	0.68–2.71	1.07	0.57–1.95
Public organization (ref.)				
Private organization	1.30	0.72–2.34	1.27	0.76–2.11
Number of care events during the day	1.03**	**1.01**–**1.05**	1.03**	**1.01**–**1.04**
Mean CMI of clients (percent)	1.05*	**1.00**–**1.09**	1.02	0.99–1.06
Breaktime (minutes)	0.99	0.97–1.00	0.98*	**0.97**–**1.00**
Recovery, good (ref.)				
Recovery, medium	1.99*	**1.06–3.86**	1.26	0.75–2.12
Recovery, poor	4.91***	**2.48**–**10.06**	1.67	0.92–3.03
Workday went as planned (ref.)				
Something disrupted the workday	5.62***	**3.25**–**9.89**	2.58***	**1.57**–**4.23**
C-statistic:	0.77		0.68	

* = *p* < 0.05, ** = *p* < 0.01, *** = *p* < 0.001

OR: Odds Ratio

CI: Confidence Interval

CMI: Case Mix Index

## Discussion

The results of our study indicate that nurses working in Finnish long-term care (assisted living facilities) have greater odds of high stress when the number of care events during the day and clients’ care needs increase. Similarly, the odds of being under high time pressure increase with the number of care events during the day and with reduced breaktime. Additionally, poor recovery from work was associated with increased stress, and something disrupting the workday was associated with both high stress and high time pressure. In terms of the organization-side variables, the participants reporting high stress and time pressure worked more often in teams with lower team autonomy.

Our findings confirm and expand on previous research using mainly subjective instruments as explanatory variables of job demands. The results align with our conceptual framework and the JD-R model [20], indicating that focusing on basic and measurable workday characteristics, such as better division of care work tasks and taking varying care needs of clients into account, can affect the perceived stress and time pressure of nurses working in long-term care. This can ultimately improve wellbeing related outcomes among nurses. The results are significant, as they are not based on only perceived variables, but on independent data derived from time measurement forms. Additionally, our results illuminate previously largely unexplored associations between the care needs of clients and nurses’ perceived wellbeing in long-term care.

As previous research has linked stress and time pressure to leaving intentions [48] and sickness absences [49], our results demonstrate that improving the division of care tasks, both in quantity (number of care events) and in quality (care needs of clients), can potentially lead to increased retention and better workforce availability of nurses, in addition to higher quality of care via more constant staff [50]. In contrast, perceived unfair division of care tasks can increase retirement intentions among older care staff, especially among nurses with low job involvement [51]. More emphasis should be placed on just and fair management of work in long-term care, which includes accounting for the relatively easy to measure number of care events and clients’ care needs, as these appear to affect the wellbeing of nurses and could ultimately increase retention and workforce availability.

Interestingly, ownership of the organization appeared to have no effect on the perceived stress or time pressure. Previous research from other Nordic countries indicates that while public organizations appear to perform better on structural quality measures, such as staffing levels (in Finland potentially extending beyond the regulated minimum), private organizations tend to score higher on processual quality, for instance individualized care [52, 53]. Our results potentially indicate that some other factors, such as lower client care needs, better leadership, or more streamlined processes might equalize the perceived stress and time pressure between private and public care organizations, regardless of lower staffing levels in private organizations. As over half of Finnish assisted living facilities are under private ownership, further research is needed on the potential differences between different organizations.

The findings suggest that nurses caring for clients with higher care needs might more often perceive high stress: a 10% increase in the average CMI of the workday’s clients increased the odds of high stress of nurses by 56%. Surprisingly, increased care needs of clients was not associated with being under high time pressure. This might indicate that sufficient time or workforce is allocated for care, regardless of individual client’s care needs, signaling the potential success of the care units to take client needs adequately into account when planning care responsibilities of nurses. Nonetheless, clients with higher care needs being perceived as more stressful among nurses can lead to lower wellbeing and work satisfaction, especially if work division among clients is not sufficient or fair. There are some options to alleviate stress and time pressure related to clients’ care needs. First, the results should be taken into account when placing clients in different care units. However, as there is only a limited number of care units per location, this may be difficult in practice. Second, in a study by Corneliusson and coauthors [50], managers of Finnish long-term care units reported task rotation as a way to control for clients’ varying care needs and reduce job strain among employees. However, this kind of rotation may also have negative effects, especially in self-organizing teams if teamwork is disrupted, which can lead to a decrease in care continuity.

The third recommendation is the use of self-organizing teams, which have seen positive outcomes in several sectors. While teamwork does not alleviate stress from clients’ care needs directly, it may lower stress in other ways. In autonomous teams employees have better possibilities to plan their work and take into account clients’ personal needs as well as employees’ individual differences [21, 54]. Our results indicated that higher team autonomy might further reduce nurses’ perceived stress or time pressure, possibly through better and more just division of care tasks. The autonomy of nurse teams has been previously associated with lower turnover via lower job demands [16]. In addition, nurses with higher team autonomy have been shown to be more engaged in their work and less likely to turnover [55]. These results suggest that nurses working in teams where they can influence their work might have less time pressure and perceive the division of care tasks among clients with varying care needs as more just. As such, the autonomy of care teams should be further explored and developed in long-term care, as it may reduce the stress and time pressure of nurses and thus increase their wellbeing.

Another notable result was the number of care events, which was associated with both stress and time pressure. In this study, the number of care events was also used as a proxy for the intensity of the care work, as it correlated strongly with the amount of care time. One direct way to influence job intensity in long-term care is staffing level legislation, which mandates a ratio of care staff to clients [56]. By increasing the number of nurses in relation to clients, job demands should decrease. However, staffing level legislation might have unintended negative consequences, as it directly increases the need for care workforce, which if not met can potentially lead to lower availability of care or to diversion of care resources from other care forms. During the study period, the staffing level legislation was in its transitory period, with 0.55 nurses per client. It has since increased to 0.65, but will likely decrease to 0.60 by 2026. Consequently, based on previous research [57, 58], the job strain of nurses in Finland should have lowered somewhat since the study period, but more research is needed to examine the effects of staffing level regulation on work-related wellbeing.

The results concerning breaktime, recovery, and workday disruptions are in line with previous research, where interruptions were linked with higher perceived nursing workload and medication errors [59] and rest breaks were associated with better physical and mental wellbeing [60]. Ensuring that nurses receive enough uninterrupted opportunities for breaks is important to enable sufficient recovery and maintaining of work ability. However, it is necessary to note that high time pressure can also directly lead to nurses not having enough time for breaks. Previous research has indicated that nurses under high time pressure might only conduct basic care [61], but our findings suggest that nurses might also reduce their own breaktime to cope with care demands and high time pressure. To prevent poor recovery and enhance the wellbeing of employees, managers should ensure that nurses have enough time to take breaks.

This study provides important insights into the role of different work characteristics and organization factors on the perceived daily stress and time pressure of nurses working in long-term care, as the effects of previously scarcely explored indirectly measured workday characteristics were demonstrated. In terms of implications for enhancing the wellbeing and retention of nurses, our findings highlight the significance of fair work division, which includes accounting for both the intensity of the care work and the clients’ care needs. These results add to the evidence on objectively or indirectly measured work demands and organizational factors affecting the wellbeing of nurses working in long-term care. Future research should explore interventions that can contribute to fair work division, help maintain work ability among nurses, and reduce stress and time pressure. In addition, more evidence is needed on the role of staffing levels in relieving the workload, and on the other hand on its wider effects on the care system.

### Limitations

Our study had a few distinct advantages. The use of separately measured variables for workday characteristics and job demands minimizes common method bias [62], which is a frequent weakness in cross-sectional studies. Next, in terms of generalizability, the sample size was decent and relatively robust and representative, with care units from both rural and urban areas, and from both the public and private care units across Finland. Last, the care needs of the clients were taken into account by using RAI assessments and the Case Mix Index, which is based on the wage-adjusted care time needed by different client groups in Finnish home care units [41].

Our study also had some limitations. The short 24-hour study period, while often used and deemed valid in previous studies [35], might influence the results through natural day-to-day variations in both the actual and perceived workloads. Next, nurses filling the time measurement forms themselves (as opposed to external observers) might affect the reliability of the results through recall bias or social desirability [63]. We aimed to mitigate these factors by using synchronous (active) time tracking and requiring starting and ending times for tasks instead of a duration. In addition, the organization of care for older people varies country by country, which can affect the generalizability of the results beyond Finland. The care needs of the clients in assisted living facilities might be higher in Finland than in other countries, due to the major role of home care in the long-term care of older people. However, the generalizability of work stressors and wellbeing has been previously reported as relatively stable among different European countries [64, 65] and even globally [66].

Next, while the study included some organizational variables, it is possible that some variability in stress or time pressure between nurses might be explained by differences between care organizations that were not measured or controlled for in this study. For example, some organizations or geographical areas could have faced difficulties in recruitment and had a lack of nursing staff, which might have affected the results. Furthermore, there were still regional COVID-19 restrictions in place during the study period, which in addition to local outbreaks could have affected some care units more than others.

Due to their positively skewed distribution, the outcome variables of stress and time pressure were categorized, which can affect the results. Consequently, sensitivity analysis was conducted with varying cutoff points for the outcome variables. The association of care needs of the clients with stress was affected by more lenient categorization, losing its statistical significance. However, with both a stricter and a more lenient categorization of high stress or time pressure, other variables remained statistically significant, retaining the direction and the overall effect of the results. Last, due to the cross-sectional design of the study, the causality and directionality of the results cannot be inferred.

## Conclusions

Our study, using indirectly measured workday characteristics, indicated that the wellbeing of nurses might be improved by better work division, through reducing job demands, dividing the workload related to clients with varying care needs, and ensuring sufficient breaktime. In addition, increasing the autonomy of teams can reduce the perceived stress and time pressure of nurses. The results highlight the responsibility the managers of care units have regarding fair work division, which promotes wellbeing, job satisfaction, and retention of nurses. Last, the use of legislative and governance tools, such as staffing level legislation, should be further researched and carefully considered. Regardless, much more must be done to increase the retention and recruitment of care staff and maintain the work ability of current nurses in long-term care for older people, which will only face increasing care demands as the population ages.

### Electronic supplementary material

Below is the link to the electronic supplementary material.

Supplementary Material 1

## Data Availability

The datasets used and analyzed during the current study are available from the corresponding author on reasonable request.

## Abbreviations

CI: Confidence interval
CMI: Case Mix Index
OR: Odds ratio
RAI: Resident Assessment Instrument
RUG: Resource Utilization Groups
SD: Standard deviation
VIF: Variance inflation factor
