# Supplementary material for: The role of workday characteristics on perceived stress and time pressure among nurses in Finnish long-term care – a cross-sectional study
Source: BMC Health Serv Res. 2024 Aug 2;24:878. doi: 10.1186/s12913-024-11294-4 (PMC11295524; doi:10.1186/s12913-024-11294-4)
Supplement: Supplementary file 1 — Supplementary Material 1 [file 12913_2024_11294_MOESM1_ESM.docx]

Supplementary file 1: Questionnaires used in the study.

**Questions in the wellbeing survey**

| **Item** | **Scale** | **Reference** |
| --- | --- | --- |
| **Before the workday** |  |  |
| *“Have you recovered from the strain caused by the previous workday?* | 0 = *“Not at all”* to 10 = *“Fully”* |  |
| **After the workday** |  |  |
| Please assess how your workday went today: | *“[Workday] went as planned”*, *“[Workday] went nearly as planned”*, and *“Something disrupted the course of the workday”* |  |
| Stress: *“Did you feel any stress today? (Stress means the situation when a person feels tense, restless, nervous, or anxious)”* | 1 = *“Not at all”* to 5 = *“Very much”* | Elo et al. [30] |
| How much the following disturbed, worried, or burdened you: |  |  |
| Time pressure: *“I had too little time for my patients/clients”* | 1 = *“Not at all”* to 5 = *“Very much”* | Harris [31] |
| Time pressure: *“I did not have time to perform my work properly”* | 1 = *“Not at all”* to 5 = *“Very much”* | Harris [31] |
|  |  |  |

**Questions from the survey for the managers**

| **Item** | **Scale** |
| --- | --- |
| Size of the care unit (number of clients) | Numeric value |
| Is the team able to decide autonomously of the following: |  |
| 1. Work shift planning 2. Division of care work tasks 3. Recruitment of new employees 4. Use of substitute workers 5. Participation in training | 1 = *“Not at all”* to 4 = “*Team is able to make decisions autonomously”* |

**The Time Measurement form**

How to fill the form:
- Fill the workday start and end times. Write the client’s full name clearly.
- Write the start and end times of your visit with a client, and what you did with the client. It’s important to distinguish if work done with a client or not (other). Remember to fill the related actions. Example: If you’re with a client, write time class 1 and if you’re doing medicine management, write action 7. In addition, if you’re helping the client with eating, add action 4.

**Time class 1**: Direct care time (client present)

| **Actions**: You can fill multiple, separate with a comma, for example: 7,8 | | | | |
| --- | --- | --- | --- | --- |
| **1** Daily hygiene, toilet | **2** Shower, bath, sauna | **3** Dressing up, movement, turning the client | **4** Aiding with eating/heating food | **5** Nursing procedures |
| **6** Medicine/drug care and follow-up | **7** Cleaning up, making sure the environment is cozy | **8** Nursing documentation (client present) | **9** Service needs assessment, care planning | **10** Furthering functional ability and rehab, outdoors |
| **11** Guiding or helping the client with their matters (incl. IT) | **12** Helping /guiding relatives | **13** Multidisciplinary work (with the client) | **14** Guiding or briefing worker/student (client present) |  |

**Time class 2**: Direct care time (group activity)

| **Actions:** | | | |
| --- | --- | --- | --- |
| **1** Rehabilitation | **2** Helping with eating | **3** Common activity | **4** Outdoors |

**Time class 3**: Indirect care time (client not present)

| **Actions**: You can fill multiple, separate with a comma, for example: 2,5 | | | |
| --- | --- | --- | --- |
| **1** Organizing client’s services and benefits | **2** Nursing documentation | **3** Assessment of needs for services, care planning | **4** Medicine management |
| **5** Helping/guiding relatives | **6** Consultation | **7** Multidisciplinary meetings |  |

**Time class 4**: Other

| **Actions:** You can fill multiple, separate with a comma, for example: 7,8 | | | | |
| --- | --- | --- | --- | --- |
| **1** Lunch break / other break | **2** Meetings | **3** Guiding or briefing worker/student, drug-exam and evaluation | **4** Office work incl. ordering accessories | **5** Research and development -related work |
| **6** Managerial and administrative work incl. announcements | **7** Collecting tools & clothes required (incl. maintenance) | **8** Other |  |  |

**Time class 5**: Maintenance work

| **Actions:** You can fill multiple, separate with a comma, for example: 1,2 | | | |
| --- | --- | --- | --- |
| **1** Cleaning | **2** Laundry | **3** Cooking, warming, doing dishes | **4** Other maintenance work |
